# Supplementary material for: The Argyreia collinsiae species complex (Convolvulaceae): phenetic analysis and geographic distribution reveal subspecies new to science
Source: PeerJ. 2024 Oct 30;12:e18294. doi: 10.7717/peerj.18294 (PMC11531258; doi:10.7717/peerj.18294)
Supplement: Supplemental Information 1 [file peerj-12-18294-s001.docx]

**Table S1.** Descriptions and character states of the characters included in the statistical analyses.

| **No.** | **Description or character state** | ***A. collinsiae***  **“Original morphotype”** | ***A. collinsiae***  **“Large-bract morphotype”** | ***A. dokmaihom*** | ***A. versicolor*** |
| --- | --- | --- | --- | --- | --- |
|  | ***I. Quantitative characters*** | **x̄ ± SD**  **(min–max)** | **x̄ ± SD**  **(min–max)** | **x̄ ± SD**  **(min–max)** | **x̄ ± SD**  **(min–max)** |
| 1 | Leaf blade length (cm) | 7.79±2.04  (4.4–11) | 11.74±3.54  (11.74–21.1) | 18.95±1.93  (16.5–22.1) | 9.78±0.84  (8.6–10.8) |
| 2 | Leaf blade width (cm) | 7.61 ± 1.83  (4.3–10.8) | 10.59±3.14  (10.59–17.2) | 12.47±1.71  (10.85–15.1) | 8.2±0.9  (7.4–9.5) |
| 3 | Leaf basal extension length (cm) | 1.38 ± 0.69  (0.6–3.2) | 2.52±0.71  (2.52–4.1) | 2.77±0.56  (1.7–3.3) | 0.9±0.16  (0.7–1.1) |
| 4 | Petiole length (cm) | 3.99 ± 1.75  (2–7) | 4.68±1.5  (4.68–7.9) | 5.15±1.82  (3–8.4) | 3.15±1.58  (1.3–4.7) |
| 5 | Outer bract length (cm) | 2.42 ± 0.52  (1.9–3.5) | 4.29±0.94  (4.29–7.6) | 5.77±0.67  (4.4–6.1) | 3.68±0.2  (3.5–4) |
| 6 | Outer bract width (cm) | 0.85 ± 0.14  (0.7–1.1) | 2.55±0.67  (2.55–4.5) | 5.02±0.51  (4–5.3) | 1.92±0.24  (1.7–2.3) |
| 7 | Ratio of outer bract length to width | 2.84 ± 0.26  (2.45–3.33) | 1.73±0.31  (1.73–2.4) | 1.15±0.03  (1.1–1.18) | 1.94±0.23  (1.61–2.22) |
| 8 | 1^st^ inner bract length (cm) | 2.01 ± 0.31  (1.5–2.5) | 3.55±0.53  (3.55–5) | 4.23±0.56  (3.4–4.7) | 3.44±0.17  (3.3–3.7) |
| 9 | 1^st^ inner bract width (cm) | 0.8 ± 0.14  (0.6–1.1) | 2.1±0.54  (2.1–3.2) | 3.2±0.25  (2.8–3.4) | 1.68±0.22  (1.5–2) |
| 10 | Ratio of 1^st^ inner bract length to width | 2.54 ± 0.5  (2–3.43) | 1.75±0.31  (1.75–2.38 | 1.32±0.14  (1.06–1.46) | 2.07±0.2  (1.75–2.27) |
| 11 | Pedicel length (cm) | 0.99 ± 0.29  (0.5–1.5) | 0.35±0.15  (0.35–0.7) | 0.35±0.16  (0.2–0.5) | 0.26±0.09  (0.2–0.4) |
| 12 | Outer sepal length (cm) | 1.25 ± 0.13  (1–1.45) | 1.42±0.19  (1.42–1.8) | 1.37±0.1  (1.2–1.45) | 1.2±0.1  (1.1–1.3) |
| 13 | Outer sepal width (cm) | 0.98 ± 0.13  (0.8–1.3) | 1.03±0.21  (1.03–1.6) | 0.94±0.08  (0.8–1) | 0.88±0.11  (0.8–1) |
| 14 | Middle sepal length (cm) | 1.33 ± 0.14  (1.1–1.6) | 1.46±0.19  (1.46–1.8) | 1.31±0.06  (1.2–1.35) | 1.27±0.07  (1.2–1.35) |
| 15 | Middle sepal width (cm) | 1.16 ± 0.24  (0.9–1.8) | 1.17±0.2  (1.17–1.7) | 0.81±0.02  (0.8–0.85) | 0.94±0.05  (0.9–1) |
| 16 | Inner sepal length (cm) | 1.3 ± 0.11  (1.05–1.45) | 1.43±0.14  (1.43–1.75) | 1.39±0.45  (1–2.27) | 1.22±0.03  (1.2–1.25) |
| 17 | Inner sepal width (cm) | 1.1 ± 0.21  (0.8–1.65) | 1.26±0.19  (1.26–1.65) | 0.79±0.05  (0.7–0.83) | 0.97±0.04  (0.9–1) |
| 18 | Corolla length (cm) | 5.02 ± 0.7  (3.8–6.1) | 5.43±0.63  (5.43–6.6) | 6.13±0.25  (5.8–6.5) | 5.96±0.62  (5.1–6.4) |
| 19 | Width of the widest part of midpetaline band (cm) | 1.3 ± 0.24  (0.8–1.7) | 1.36±0.11  (1.36–1.5) | 1.39±0.18  (1.1–1.6) | 1.36±0.11  (1.2–1.5) |
| 20 | Filament length (cm) | 1.83 ± 0.19  (1.5–2.2) | 1.85±0.19  (1.85–2.3) | 2.05±0.14  (1.9–2.3) | 1.72±0.16  (1.5–1.9) |
| 21 | Anther length (cm) | 0.63 ± 0.05  (0.6–0.7) | 0.59±0.06  (0.59–0.7) | 0.62±0.08  (0.5–0.7) | 0.6±0  (0.6–0.6) |
| 22 | Non-glandular trichome stalk length (mm) | 0.08 ± 0.02  (0.05–0.12) | 0.1±0.04  (0.1–0.23) | 0.11±0  (0.11–0.11) | 0.19±0.03  (0.16–0.24) |
| 23 | Non-glandular trichome stalk width (mm) | 0.08 ± 0.01  (0.07–0.11) | 0.08±0.01  (0.08–0.1) | 0.08±0.02  (0.06–0.1) | 0.09±0.01  (0.08–0.1) |
| 24 | Apical cell of non-glandular trichome length (mm) | 1.22 ± 0.16  (0.84–1.49) | 1.37±0.31  (1.37–1.89) | 1.51±0.26  (1.23–1.75) | 1.05±0.17  (0.82–1.2) |
| 25 | Apical cell of non-glandular trichome width (mm) | 0.05 ± 0.01  (0.04–0.07) | 0.05±0.01  (0.05–0.05) | 0.05±0.01  (0.04–0.06) | 0.05±0.01  (0.04–0.06) |
| 26 | Epidermal cell length of staminal trichome (mm) | 0.03 ± 0  (0.03–0.04) | 0.03±0  (0.03–0.04) | 0.03±0  (0.03–0.03) | 0.03±0.01  (0.03–0.04) |
| 27 | Apical cell of glandular trichome diameter (mm) | 0.07 ± 0.01  (0.07–0.08) | 0.07±0.03  (0.07–0.1) | 0.06±0.01  (0.06–0.07) | 0.06±0.01  (0.06–0.07) |
|  | ***II. Qualitative characters*** |  |  |  |  |
| 28 | Leaf apex: 1 = Acute; 2 = Acuminate | Acute or Acuminate | Acute or Acuminate | Acuminate | Acute or Acuminate |
| 29 | Leaf shape: 1 = Very widely ovate; 2 = Widely ovate; 3 = Ovate | Very widely ovate to widely ovate | Very widely ovate to widely ovate | Very widely ovate to ovate | Very widely ovate to widely ovate |
| 30 | Adaxial leaf surface; 1 = Puberulent; 2 = Scabrous-strigose; 3 = Strigose | Puberulent | Puberulent | Strigose | Scabrous-strigose |
| 31 | Adaxial leaf surface color: 1 = Greyish; 2 = Brown | Greyish | Greyish | Brown | Greyish |
| 32 | Density of inflorescence: 1 = Lax; 2 = Dense | Lax | Lax | Dense | Lax |
| 33 | Smell: 1 = Absent; 2 = Present | Absent | Absent | Present | Absent |
| 34 | Persistence of bract: 1 = Caducous; 2 = Persistent | Caducous | Persistent | Persistent | Persistent |
| 35 | Hairs on outer sepals: 1 = Absent; 2 = Present | Absent or Present | Absent | Absent | Present |
| 36 | Hairs on middle sepals: 1 = Absent; 2 = Present | Absent | Absent | Absent | Present |
| 37 | Hairs on midpetaline bands: 1 = Absent; 2 = Present | Absent | Absent | Absent | Present |
| 38 | Lip color: 1 = Purple; 2 = Pale purple; 3 = White | Purple | Purple | White | Pale purple |
| 39 | Long stalked glandular trichome on the base of stamens: 1 = Absent; 2 = Present | Absent | Absent | Absent | Present |
| 40 | Branched non-glandular trichome on the base of stamens: 1 = Absent; 2 = Present | Absent | Absent | Present | Absent |

**Table S3.** The environmental variables used for modeling and their variance inflation factor (VIF) values.

| *A. collinsiae* “Original morphotype” | | |
| --- | --- | --- |
| Coded | Bioclimatic variables | VIF |
| bio6 | Min Temperature of Coldest Month | 3.06 |
| bio9 | Mean Temperature of Driest Quarter | 4.82 |
| bio14 | Precipitation of Driest Month | 4.29 |
| bio18 | Precipitation of Warmest Quarter | 1.11 |
| *A. collinsiae* “Large-bract morphotype” | | |
| Coded | Bioclimatic variables | VIF |
| bio2 | Mean Diurnal Range (Mean of monthly (max temp - min temp)) | 1.48 |
| bio4 | Temperature Seasonality (standard deviation ×100) | 7.91 |
| bio10 | Mean Temperature of Warmest Quarter | 7.83 |
| bio13 | Precipitation of Wettest Month | 4.99 |
| bio14 | Precipitation of Driest Month | 2.59 |
| bio17 | Precipitation of Driest Quarter | 3.02 |
| bio18 | Precipitation of Warmest Quarter | 3.40 |

**Table S4.** Cross-validation testing and training data evaluation scores of ensemble models of the two *A. collinsiae* morphotypes.

| OTUs | Testing data | | Training data | |
| --- | --- | --- | --- | --- |
|  | TSS | AUC | TSS | AUC |
| *A. collinsiae* “Original morphotype” | 0.825 | 0.916 | 0.959 | 0.994 |
| *A. collinsiae* “Large-bract morphotype” | 0.893 | 0.973 | 0.913 | 0.988 |
