# Supplementary material for: The Argyreia collinsiae species complex (Convolvulaceae): phenetic analysis and geographic distribution reveal subspecies new to science
Source: PeerJ. 2024 Oct 30;12:e18294. doi: 10.7717/peerj.18294 (PMC11531258; doi:10.7717/peerj.18294)

**Figure S1.** FAMD results showing the contributions of the 40 study characters towards the first- and second-dimensions. The dashed lines represent the threshold value generated by the algorithm.


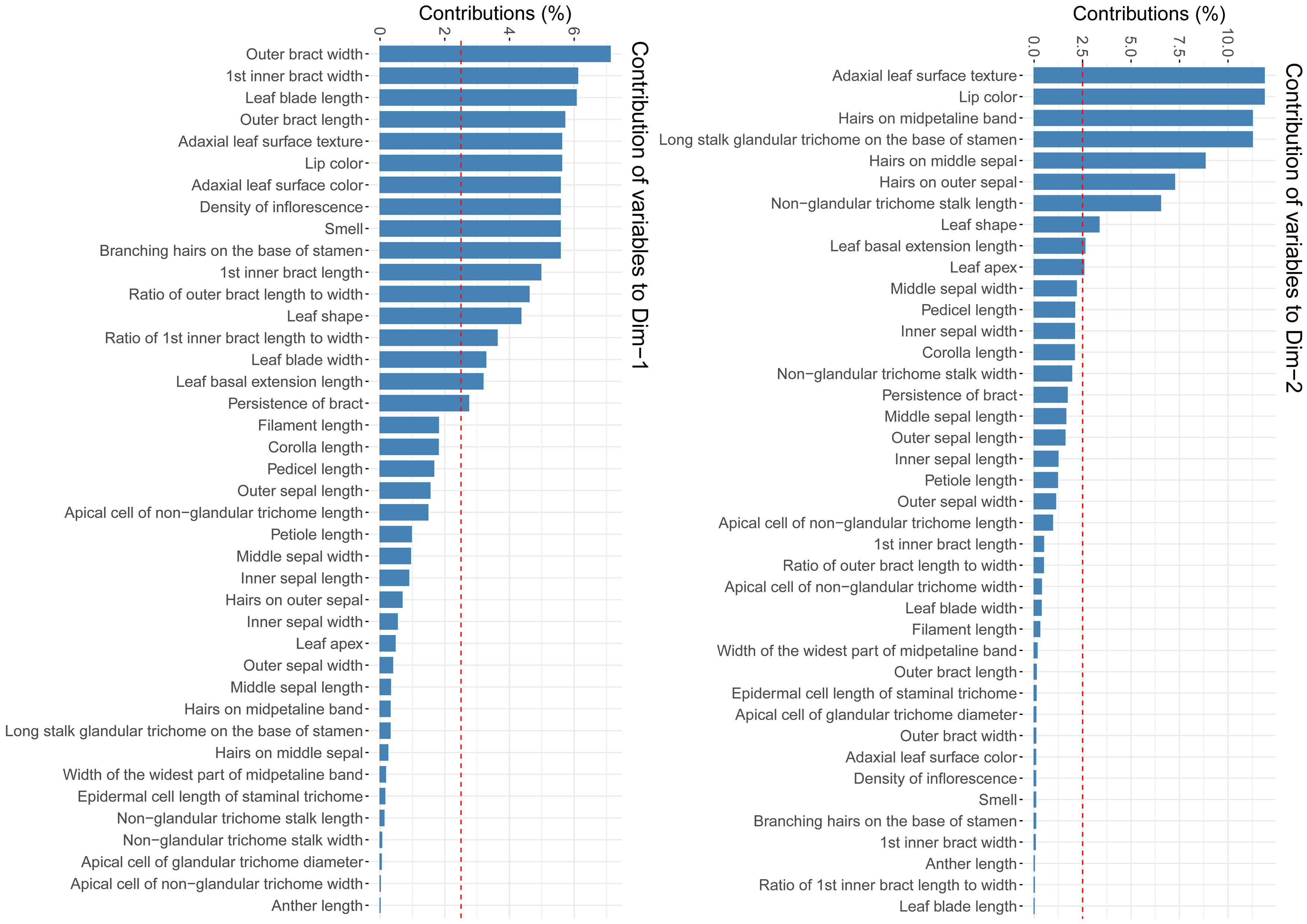


**Figure S2.** Box plot graphs showing differences between OTUs for the 10 significant morphological characters based on FAMD analysis: leaf blade length (A), leaf blade width (B), leaf basal extension length (C), outer bract length (D), outer bract width (E), ratio of outer bract length to width (F), 1st inner bract length (G), 1st inner bract width (H), ratio of 1st inner bract length to width (I) and non-glandular trichome stalk length (J). Abbreviations: ACL, large-bract *A. collinsiae* morphotype; ACS, original *A.* *collinsiae* morphotype; ADH, *A. dokmaihom*; AVC, *A. versicolor*.


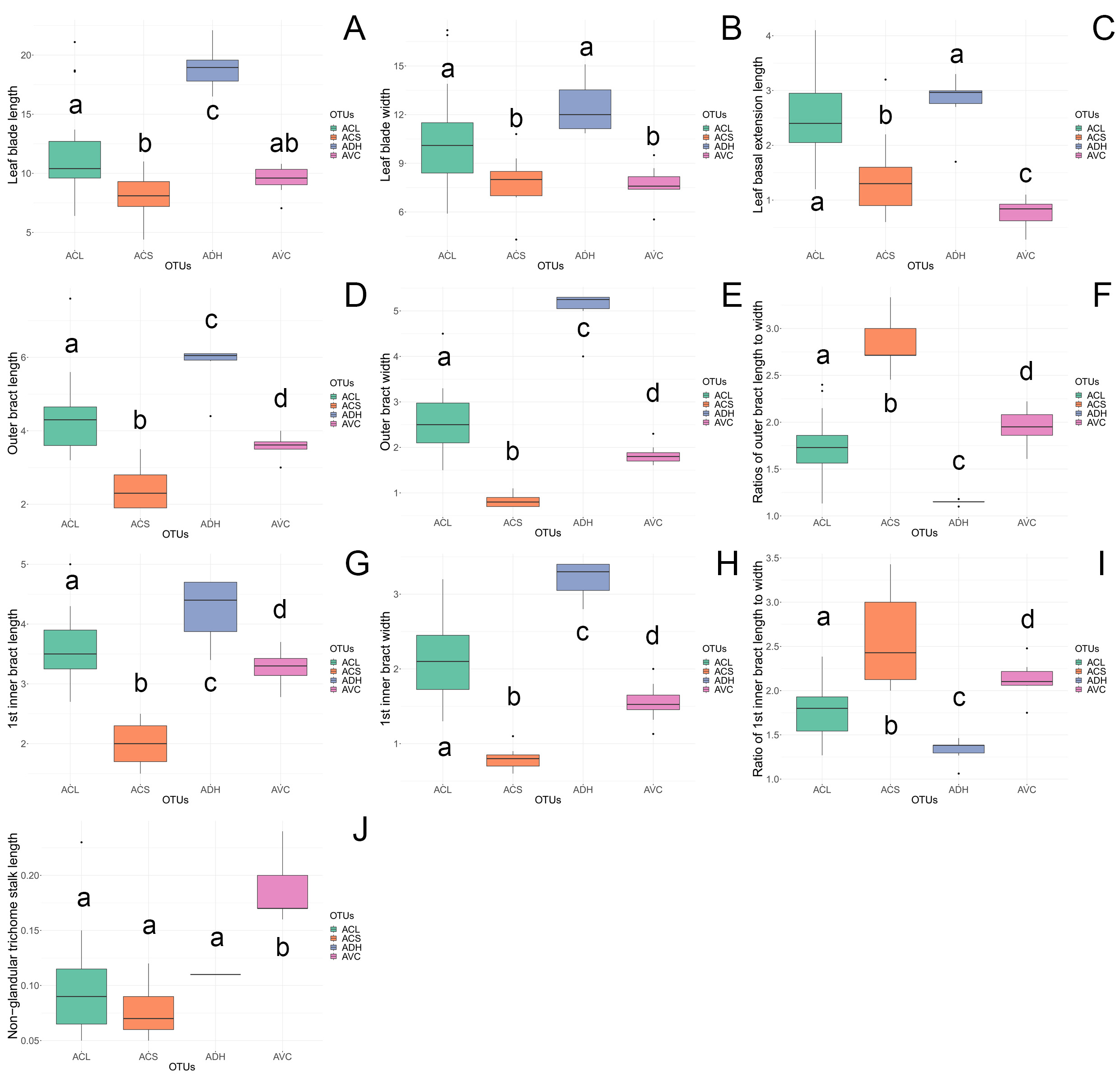

Supplement: Supplemental Information 2 [file peerj-12-18294-s002.docx]
